# Neuroticism and Aggressive Behavior among Left-Behind Children: The Mediating Roles of Interpersonal Sensitivity and Bullying Victimization

**DOI:** 10.3390/ijerph191711072

**Published:** 2022-09-04

**Authors:** Yinghan Dong, Fangfang Liu, Yingjie Jiang, Siyuan Wei

**Affiliations:** School of Psychology, Northeast Normal University, Changchun 130024, China

**Keywords:** left-behind children, neuroticism, interpersonal sensitivity, bullying victimization, aggressive behavior

## Abstract

When children are “left behind”, aggressive behavior is a common manifestation of problem behaviors, and several previous studies suggested that neuroticism has characteristics such as oversensitivity and impulsivity, which may be important predictors of aggressive behavior. However, the mediating mechanisms underlying this relationship are unknown. This study is designed to analyze how neuroticism leads to left-behind children’s aggressive behaviors through mediators of interpersonal sensitivity and bullying victimization. A sample of 1478 Chinese children (67.72% left-behind children; 37.28% non-left-behind children) through whole-class contact and voluntary participation completed measurements of neuroticism, interpersonal sensitivity, bullying victimization, and aggressive behavior. Findings from the mediation analysis show that interpersonal sensitivity and bullying victimization could mediate the relationship between neuroticism and aggressive behavior among left-behind children separately and sequentially. These findings suggest helpful ways to reduce the aggressive behaviors of left-behind children by decreasing interpersonal sensitivity and bullying victimization.

## 1. Introduction

“Left-behind children” refers to children under 18 years old who stay in their hometowns and are cared for by one of their parents, other relatives, neighbors, etc., because one or both parents have migrated to an urban area to work for more than three months [1]. Unprecedented migration among families from rural to urban areas and the inability to live with their children has caused a series of mental disorders or educational problems among left-behind children [2]. Long-term forced separation breaks normal interpersonal communication between children and parents and easily leads to neglectful behavior of parents in supervising the left-behind children [3]. Therefore, compared with non-left-behind children, left-behind children are more prone to externalization problems, such as aggressive behavior [4]. Aggressive behavior is defined as “any behavior that intentionally harms or injures others, causes physical or psychological harm to others and is not recognized by social norms” [5]. Aggressive behavior in childhood may be an effective predictor of violent and dangerous behaviors in adolescence and adulthood [6]. Therefore, there exists an urgent need to explore the antecedents of left-behind children’s aggressive behavior to provide empirical support for prevention and psychological interventions.

The general aggressive model (GAM) has the advantage of taking into account the combined effects of individual and environmental factors on aggressive behavior [5]. However, most current research on aggressive behavior focuses on the role of various factors separately and ignores the interaction between individuals and their surroundings [7]. Individuals are exposed to different risk events under the same conditions, according to the organism–environment interaction model, and individual factors are especially important in determining what experience an individual will have [8]. Therefore, based on the GAM and the organism–environment interaction model, this study will jointly investigate the influencing mechanism of aggressive behavior in left-behind children. According to the GAM, personality is an important antecedent variable of aggressive behavior [5,9]. As a key variable in understanding personality traits related to aggressive behavior, neuroticism can positively predict aggressive behavior [10,11]. However, few studies have explored the influence of neuroticism on aggressive behavior. This study focuses on the relationship between left-behind children’s neuroticism and aggressive behavior, as well as the potential mediating mechanisms underlying this relationship.

### 1.1. Neuroticism and Aggressive Behavior

Neuroticism reflects an individual’s emotional stability and is closely associated with emotional responses [12]. According to Eysenck’s biological theory of personality, neuroticism is related to higher psychobiological reactivity when confronted with frustration and higher sympathetic arousal, thus neuroticism often follows a susceptibility to stress, inefficient ways of dealing with frustration, and an inability to control urges [13,14]. Additionally, the neuropsychological model suggests that the higher the level of neuroticism is, the fewer the psychological resources available to the individual. As a result, the individual’s self-regulatory function is also constrained and tends to overperceive stimuli as threats to themselves, which leads to aggressive behavior, regardless of the consequences of the behavior [15]. Empirical evidence also supports this view. Previous research has shown that high levels of neuroticism are associated with higher levels of aggressive behavior [10]. Generally, individuals with high neuroticism tend to experience highly negative emotions, such as anxiety, anger, and less tolerance of frustration [16]. Furthermore, cognitive neuroscience research also showed that the sensitive perception of frustration and negative emotions could activate parts of the amygdala and anterior cingulate gyrus to weaken the individual’s inhibitions, which is considered closely related to aggressive behavior [17]. Thus, highly neurotic individuals are more likely to exhibit aggressive behavior.

In summary, those with high neuroticism are more likely to be aggressive, both in terms of personal traits and self-regulation. Compared with non-left-behind children, left-behind children have a higher level of neuroticism [18]. Accordingly, neuroticism may be significantly and positively associated with left-behind children’s aggressive behavior.

### 1.2. The Mediating Role of Interpersonal Sensitivity

Interpersonal sensitivity is a condition of low self-esteem and high psychological inferiority that manifests as being uncomfortable in public, hypersensitive, inferior, and introverted, as well as being prone to evaluate oneself negatively and a marked difficulty in establishing intimate relationships with others [19]. Previous evidence indicated that neuroticism can positively predict interpersonal sensitivity [20,21]. Individuals with high neuroticism tend to experience more negative emotions, and information consistent with the type of emotion is more likely to be recalled [22]. Specifically, individuals with high neuroticism are more prone to recall negative information, and accordingly, they may have more negative perceptions. Individuals exposed to these negative influences interpret ambiguous and neutral information as a rejection of themselves, causing them to become timid, inferior, and leading to increased interpersonal sensitivity [23]. Individuals who are highly neurotic in the group of left-behind children are more likely to experience negative emotions, which further increases their interpersonal sensitivity.

In addition, some studies have demonstrated that people with strong interpersonal sensitivity are thought to be more likely to engage in aggressive behavior. For example, Liu, Chen, and Liu [24] showed that, compared with individuals with low interpersonal sensitivity, individuals with high interpersonal sensitivity had more negative emotional experiences and coping styles and showed more aggression. First, when interacting with others, highly interpersonal and sensitive individuals tend to overinterpret others’ words or actions and are more sensitive to rejection, criticism, and interpersonal conflict, which leads to aggressive behaviors. Second, from the perspective of cognitive style, highly interpersonally sensitive individuals are inclined to adopt a negative attribution approach to interpreting problems and misinterpreting initially neutral social cues as opposition and rejection of themselves, leading to errors in assessing and judging problems and then engaging in aggressive behaviors to solve problems. This is also consistent with the cognitive model, which suggests that negative beliefs about ourselves may be combined with threatening evaluations of others, such as believing that others are untrustworthy, thereby triggering aggressive behavior [25]. Thus, whether from the characteristics or the cognitive style of interpersonal sensitivity, high interpersonal sensitivity leads to aggressive behavior.

Accordingly, based on prior empirical and theoretical evidence, it is inferred that interpersonal sensitivity may be a mediating variable between neuroticism and aggressive behavior among left-behind children.

### 1.3. The Mediating Role of Bullying Victimization

Additionally, why do some left-behind children develop healthily and positively, while others exhibit problematic behaviors frequently? Bullying victimization may be a key variable. Zhang et al. [26] found that approximately 31.6% of rural left-behind children are frequently bullied, significantly higher than non-left-behind children in the same region. Bullying victimization refers to an individual suffering from peer’s strong deliberate verbal or physical assault, property violations, and interpersonal harm [27].

Studies have noted that being bullied persists in children over time [28], which indicates that it is influenced by stable personality traits. Some studies have found a significant positive correlation between neuroticism and victimization [29]. The characteristics of high neuroticism make it difficult to form positive relationships with others, and therefore, individuals cannot receive strong social support from peers, resulting in a lower status in the peer group. Except for a tiny number of individuals at the top of the group pyramid, the majority of peers regularly bully children whose status is lower than their own to enhance or retain their existing status [30]. Therefore, these low-status and highly neurotic children are more likely to be “favored” by some bullies.

Ample studies have found that bullying victimization could significantly predict aggressive behavior [31,32]. Combined with previous theoretical and empirical studies, being bullied impacts an individuals’ aggressive behavior in three ways. First, in behavioral learning, bullied individuals’ violent experiences provide a basis for later aggressive behaviors, as children acquire the means and power of aggression [33]. Second, in emotional affect, according to general stress theory, people tend to be exposed to negative information after suffering negative events, such as bullying victimization, and are prone to produce negative emotions, such as anger and jealousy. Therefore, bullied children may relieve pressure and vent their emotions by attacking others [34]. Third, in terms of cognitive development, some researchers have pointed out that bullying victimization may elicit hostile expectations and produce cognitive biases. Simultaneously, bullying victimization causes children to easily form aggressive patterns, which may lead to aggressive behavior [35]. In summary, bullying victimization is expected to mediate the relationship between neuroticism and aggressive behavior among left-behind children.

### 1.4. The Chain Mediating Role of Interpersonal Sensitivity and Bullying Victimization

Individuals with different characteristics have different coping styles when faced with the same event, according to the organism–environment interaction model, so their future environmental encounters must also be different [8]. Furthermore, previous studies also indicated that children’s characteristics may contribute to bullying victimization [36]. The guiding assumption proposes that some traits, such as excessive obedience, withdrawal, and sensitivity, may become the fuse or reinforcers of being bullied [37]. A comprehensive review of previous studies on the characteristics of bullied children found that high interpersonal sensitivity is a typical attribute of bullied children [38]. The interpersonal sensitivity characteristics of introversion and timidity, nervousness and anxiety, behavioral withdrawal, and hypersensitivity to external stimuli are not conducive to peer interaction, making children with high interpersonal sensitivity more likely to experience interpersonal distress and be isolated when in conflict with others, thus increasing their likelihood of being bullied. This also precisely reflects the interaction between the psychological condition and behavioral outcomes of “positive people becoming more positive and negative people becoming more negative”. The separation of parent/child relationships causes left-behind children to focus more on peer relationships. Positive peer relationships protect children’s healthy development by increasing well-being and security [39] and mitigating the negative effects of parent/child relationships on children [40]. Therefore, interpersonal sensitivity is likely to cause left-behind children to be bullied. Based on the above literature review, one can speculate that interpersonal sensitivity and bullying victimization mediate the relationship between neuroticism and aggressive behavior among left-behind children (H4).

### 1.5. The Present Study

**Figure 1:**
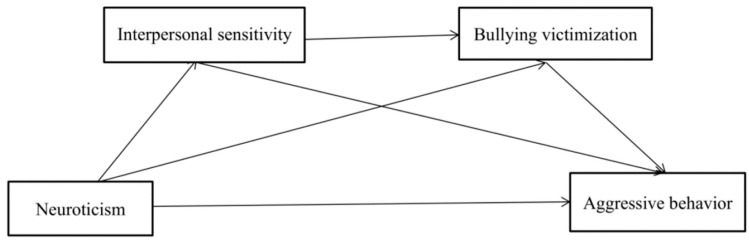
A multiple mediation model of the association between neuroticism and aggressive behavior.

Aggressive behavior among Chinese left-behind children has always been the focus of social attention. However, research on the possible mechanism of how children’s neuroticism influences their aggressive behavior remains relatively sparse. Therefore, the present study, based on the GAM and the individual–environment interaction model, aims to explore the mediating role of interpersonal sensitivity (individual factor) and bullying victimization (environmental factor) between neuroticism and aggressive behavior among Chinese left-behind children. The proposed multiple mediation model is depicted in Figure 1. According to research, left-behind children have higher levels of interpersonal sensitivity and bullying victimization [26,41], as well as more problematic behaviors such as aggression, than non-left-behind children. Therefore, does the relationship between neuroticism and aggressive behavior differ depending on the group? As a result, a cross-group model comparison was conducted within two groups of left-behind children and non-left-behind children. The following hypotheses were proposed:

**Hypothesis** **1** **(H1).** *Neuroticism might be positively associated with aggressive behavior among left-behind children.*

**Hypothesis** **2** **(H2).** *Interpersonal sensitivity mediates the link between neuroticism and aggressive behavior among left-behind children.*

**Hypothesis** **3** **(H3).** *Bullying victimization may play a mediating role between neuroticism and aggressive behavior among left-behind children.*

**Hypothesis** **4** **(H4).** *A serial path from interpersonal sensitivity to bullying victimization might mediate the link between neuroticism and aggressive behavior among left-behind children.*

**Hypothesis** **5** **(H5).** *The variables’ relationships are different between left-behind and non-left-behind children.*

## 2. Materials and Methods

### 2.1. Participants

The participants were grade 4 of primary to grade 3 of middle school students from Shanxian, Shandong Province. The survey was conducted in December 2020. A total of 1478 children completed the questionnaire; of these children, 150 completed the questionnaire invalidly (e.g., the same responses to all items) and 113 did not complete the questionnaire. After excluding participants with invalid and missing data, the final sample size was 1215, among which 762 (67.72%) were left-behind children and 453 (37.28%) were non-left-behind children. They ranged in age from 9–16 years. Among left-behind children, the average age was 12.62 (*SD* = 1.46), 379 were boys (49.74%) and 383 were girls (50.26%), 273 were elementary school students (35.83) and 489 (64.17%) were middle school students, and 353 children had one parent out (46.33%) and 409 children had both parents away (53.67%). The mean age of the non-left-behind children was 12.51 (*SD* = 1.51), 206 were boys (45.47%) and 247 were girls (54.53%), and 199 were elementary school students (43.93%) and 254 were middle school students (56.07%). The study design was approved by the Human Research Ethics Committee of Northeast Normal University.

### 2.2. Procedure

With the permission of the school, parents, and students, a web-based questionnaire was administered in a group setting in the school computer room. A specially trained teacher served as the main tester, and all testers were given special training on the instructions, content, and precautions prior to the test. The questionnaires took approximately 15 min to complete.

### 2.3. Measures

#### 2.3.1. Neuroticism

Neuroticism was assessed with the neuroticism subscale of the NEO-FFI modified by Costa and McCrae [42], which has been validated in Chinese adolescents and adults [43]. The neuroticism subscale includes 12 items, and four of them are scored in reverse. Children rated items on a five-point Likert scale (1 = strongly disagree, 5 = strongly agree). A higher score indicated a higher level of neuroticism. Cronbach’s α in the current study was 0.77, and the questionnaire structure validity was good: χ^2^/df = 6.72, CFI = 0.92, IFI = 0.92, NFI = 0.91, RMSEA = 0.07.

#### 2.3.2. Interpersonal Sensitivity

Interpersonal sensitivity was measured by the interpersonal sensitivity subscale of the symptom self-rating scale revised by Wang in 1984 [44]. The interpersonal sensitivity subscale consists of 9 items and is rated on a five-point Likert scale from “none” to “severe”, with higher scores indicating higher interpersonal sensitivity. In this study, Cronbach’s α was 0.83, and the questionnaire had good construct validity: χ^2^/df = 5.42, CFI = 0.96, IFI = 0.92, NFI = 0.92, and RMSEA = 0.07.

#### 2.3.3. Bullying Victimization Scale

Bullying victimization was measured by a 5-item scale adapted from Solberg and Olweus [45]. The children rated experiences on a four-point Likert scale (0 = none, 1 = 1 or 2 times, 2 = approximately 3 or 4 times, 3 = more than 4 times), and the sum of the scores represented the degree of bullying victimization. Cronbach’s α in the current study was 0.83, and the scale provided good validity: χ^2^/df = 7.28, CFI = 0.95, IFI = 0.96, NFI = 0.91, and RMSEA = 0.07.

#### 2.3.4. Aggressive Behavior

The Buss-Perry aggression scale was adapted [46], which has 29 items and consists of four dimensions: physical aggression, verbal aggression, anger, and hostility. Combining previous research and the reality of left-behind children, two dimensions were selected, verbal and physical aggression, to measure the level of the left-behind children’s aggressive behavior [47]. There were 7 items in total and a five-point scale (1 = no, 5 = severe), with higher scores indicating greater aggression. Cronbach’s *α* was 0.82, and the structure validity of the scale was good: χ^2^/df = 5.45, CFI = 0.96, IFI = 0.95, NFI = 0.91, RMSEA = 0.06.

### 2.4. Data Analyses

All the data were analyzed using SPSS 22.0. First, common method biases were tested by factor analysis, and the results showed 6 factors with characteristic roots over 1. The first factor to explain the variance was 24.25%, which indicated that the present study had no significant problem with common method biases. Second, descriptive statistics and Pearson’s correlation analyses were employed to present the relations among variables. Third, the hypothesized model was analyzed using model 6 of the SPSS macro PROCESS. Then, 5000 bootstrap samples were used to estimate the significance of the indirect effects. If the 95% confidence intervals (CIs) do not include zero, the effects are significant.

## 3. Results

### 3.1. Descriptive Statistics and Correlation Analysis

Table 1 presents the means, standard deviations, and correlations among the four variables. The results exhibit significant positive correlations between neuroticism, interpersonal sensitivity, bullying victimization, and aggressive behavior.

### 3.2. Mediation Analyses

**Figure 2:**
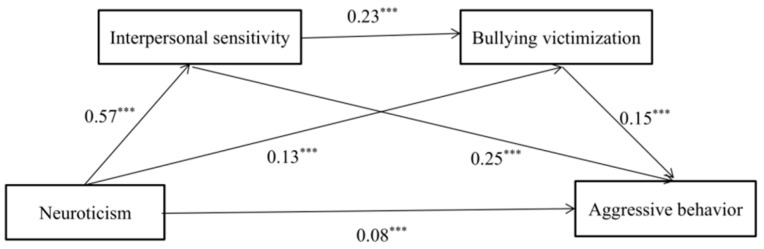
Results of mediation analysis for aggressive behavior among left-behind children. Standardized coefficients are presented. ***Note.*** *** *p* < 0.001. *n* = 762.

The structural model comprises neuroticism as a predictor factor, interpersonal sensitivity as the first mediator, bullying and victimization as the second mediator, and aggressive behavior as the outcome variable. Age and gender were regarded as control variables. Table 2 presents the results of the regression analysis, and Figure 2 clearly displays all relationships. Table 2 demonstrates that among left-behind children, neuroticism was positively linked with aggressive behavior, interpersonal sensitivity, and bullying victimization (*β* = 0.27, *p* < 0.001; *β* = 0.57, *p* < 0.001; *β* = 0.13, *p* = 0.004). Interpersonal sensitivity was positively and significantly linked to bullying victimization and aggressive behavior (*β* = 0.23, *p* < 0.001; *β* = 0.25, *p* < 0.001). In addition, after incorporating interpersonal sensitivity and bullying victimization into the regression equation, neuroticism was still significantly associated with aggressive behavior (*β* = 0.08, *p* = 0.002). Finally, bullying victimization was positively and significantly related to aggressive behavior (*β* = 0.15, *p* < 0.001). Table 3 specifically shows the total indirect effects. The indirect effect of neuroticism on aggressive behavior via the separated mediation of interpersonal sensitivity and bullying victimization (95% CI [0.10,0.19]; 95% CI [0.01,0.03]) was significant. Additionally, the specific indirect effect of neuroticism on aggressive behavior via the serial mediation of interpersonal sensitivity and bullying victimization (95% CI [0.01,0.04]) was significant.

### 3.3. Model Comparison of Left-Behind and Non-Left-Behind Children

**Figure 3:**
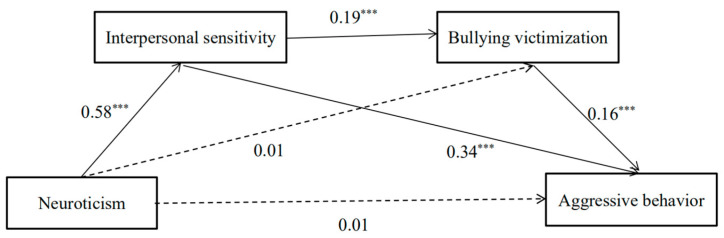
Results of mediation analysis for aggressive behavior among non-left-behind children. Standardized coefficients are presented. ***Note.*** *** *p* < 0.001. *n* = 453.

To test whether the model differed between the left-behind and non-left-behind children, the chain mediation model was also tested in the non-left-behind children, and the results are shown in Table 2 and Table 3. Age and gender were controlled as covariates. First, neuroticism was not a significant predictor of aggressive behavior among non-left-behind children, with a path coefficient of 0.01 (*p* = 0.96), but it was significant in the left-behind children, with a path coefficient of 0.08 (*p* < 0.001). Second, neuroticism was not a significant positive predictor of bullying victimization in the non-left-behind children group, with a path coefficient of 0.01 (*p* = 0.90), but neuroticism predicted bullying victimization in the left-behind children, with a path coefficient of 0.13 (*p* = 0.004). The final model is shown in Figure 3.

## 4. Discussion

### 4.1. The Direct Link between Neuroticism and Aggressive Behavior

The current study finds that the neuroticism of left-behind children directly and positively predicts their aggressive behavior, while this pathway is not significant in the non-left-behind children group, which supports Hypotheses 1 and 5. This finding supports previous research on the impact of an individual’s personality on problem behaviors [48]. However, parental presence provides more behavioral guidance and support for non-left-behind children, and these factors may play a protective role in the relationship between neuroticism and aggression. Thus, neuroticism is not a significant predictor of aggression among non-left-behind children.

### 4.2. Mediating Role of Interpersonal Sensitivity

Findings from the current study support Hypothesis 2, suggesting that interpersonal sensitivity plays a mediating role in the relationship between neuroticism and aggressive behavior. Neuroticism is an essential factor in shaping children’s interpersonal sensitivity. Highly neurotic children who are irritable, depressed, emotionally reactive, and impulsive may be more likely to be rejected in peer interactions [23]. Therefore, highly neurotic children are prone to have higher interpersonal sensitivity levels.

Moreover, interpersonal sensitivity can positively predict aggressive behavior, which is consistent with Western findings [49]. Aggression may be caused by an individual’s hostile interpretation of a situation or cue. The more reasonably and correctly a person can interpret these cues, the lower the possibility of aggression. However, children with high interpersonal sensitivity tend to misinterpret others’ words or behaviors, so they easily show more aggressive behaviors in the face of stimulating situations.

### 4.3. The Mediating Role of Bullying Victimization

Neuroticism can influence left-behind children’s aggressive behavior by influencing their being bullied, which is consistent with problem behavior theory [50]; this result also supports Hypothesis 3. Most highly neurotic left-behind children with lower social skills and self-esteem are more prone to experience anxiety and have problems communicating, so they are more likely to be bullied and isolated.

Furthermore, victims may be more prone to aggressive behaviors than bullies because children’s experiences of facing negative stimuli, such as bullying victimization, increase psychological pressure, which will urge them to engage in dangerous or illegal behaviors to deal with tension [50]. Non-left-behind children, however, can be detected by their parents when they are emotionally unstable and receive appropriate support and reassurance, effectively preventing bullying.

### 4.4. The Mediating Role of Interpersonal Sensitivity and Bullying Victimization

The results further demonstrate that interpersonal sensitivity and bullying victimization mediate the relationship between neuroticism and aggressive behavior among left-behind children, which supports Hypothesis 4. High neuroticism contributes to high interpersonal sensitivity. Meanwhile, high interpersonal sensitivity usually causes poor peer relationships, which is a risk factor for predicting bullying victimization [51]. Due to parent/child separation, peer relationships play an important role in the socialization of left-behind children. Therefore, children with high interpersonal sensitivity do not receive the support and encouragement of their peers when confronted with stressful situations, resulting in a lower status in their peer group. Furthermore, children tend to consolidate their existing status by bullying children whose status is lower than their own, so children with higher interpersonal sensitivity are more likely to become victims [30]. This multiple mediation model demonstrates that neuroticism influences an individual’s aggressive behavior via interpersonal sensitivity and bullying victimization.

### 4.5. Limitations and Implications

There are several limitations to the current study. First, this study only focuses on the predictive effect of the neurotic personality of left-behind children on interpersonal sensitivity, bullying, and aggressive behavior, but the roles of personality dimensions, such as friendliness and openness, have not been investigated. Further research on these dimensions can be carried out in the future to provide effective suggestions. Second, the study is dependent on self-reported data collected from left-behind children, which may involve common method variance. In the future, objective data can be obtained through the evaluation of others. Third, this study adopts a cross-sectional design, and causal inferences cannot be established. The results support the theoretical predictions, and the study provides useful support for the general theory. Future research can be combined with longitudinal research to investigate the causal directions of the studied associations. Finally, during the testing process, we did not assess well whether the subjects had been bullied and whether they received corresponding support previously, nor did we classify the bullying situation, so this confounding factor cannot be ruled out. Future research can fully explore the relationships with various variables such as aggression on the basis of refining the types of bullying victims.

Despite these limitations, the findings have important implications for guiding parents and teachers in reducing the negative impact of left-behind children’s high neuroticism on aggressive behavior. For parents, first of all, parents should establish a correct concept of education and realize that family education is not only the safety and material satisfaction of children, but more importantly, the development of a sound personality. Furthermore, parents should pay attention to providing child with positive emotional responses. Positive responses are conducive to strengthening left-behind children’s self-expression and interpersonal skills, which in turn promotes social adaptation to change their sensitivity, low self-esteem, and impulsivity, reducing the likelihood of their aggressive behavior. Second, the results of this study indicate that interpersonal sensitivity and bullying are significant risk factors for aggressive behavior; parents can choose appropriate times to communicate directly with their children about their daily lives using the internet, allowing them to talk about their gains and difficulties at school and at home, rather than simply asking about their physical and academic situations, discovering their needs, providing guidance and support, and also easing their interpersonal sensitivity in their communication to avoid bullied or aggression. For teachers, first, teachers should understand children’s personality characteristics and actively pay attention to highly neurotic children. Second, the multiple mediation model demonstrates that the interpersonal sensitivity of left-behind children may influence being bullied, which then result in aggressive behavior. On the one hand, educators should pay close attention to the interpersonal sensitivity of left-behind children. When conducting mental health education, they can use group counseling and club activities to cultivate students’ interpersonal skills and guide students to establish good peer relationships and improve their interpersonal skills. On the other hand, teachers should be aware of the occurrence of bullying in real time and utilize different interventions for different types of bullying. Finally, educators need to pay more attention to the behavior and mental health status of bullied left-behind children, promptly detect problem behaviors, and implement targeted interventions. Teachers should assist students in getting rid of their emotions and make appropriate attributions during the intervention of bullied children, to avoid the vicious circle of being bullied and aggression caused by hostility or revenge.

## 5. Conclusions

This study adds to our understanding of the underlying mechanisms between neuroticism and aggressive behavior in left-behind children. Furthermore, significant pathways leading from neuroticism to interpersonal sensitivity, bullying victimization, and aggressive behavior highlight the complex relationships between these variables. We hope that future research will continue to explore how and when different personal and situational factors may jointly influence aggressive behaviors among left-behind children.

## Figures and Tables

**Table 1:** Descriptive statistics and correlations between variables (*n* = 1215).

	Variables	*M* ± *SD*	1	2	3	4	5	6
1	Gender	0.50 ± 0.50 (0.55 ± 0.50)	1					
2	Age	12.62 ± 1.46 (12.51 ± 1.51)	−0.05 (−0.06)	1				
3	Neuroticism	2.72 ± 0.65 (2.63 ± 0.69)	0.07 (0.15 **)	−0.08 (0.05)	1			
4	Interpersonal sensitivity	1.71 ± 0.70 (1.67 ± 0.73)	0.04 (0.04)	−0.11 ** (−0.02)	0.56 *** (0.51 ***)	1		
5	Bullying victimization	0.61 ± 0.70 (0.58 ± 0.70)	−0.02 (−0.05)	−0.43 *** (−0.40 ***)	0.25 *** (0.10 *)	0.29 *** (0.20 ***)	1	
6	Aggressive behavior	1.42 ± 0.55 (1.39 ± 0.55)	−0.13 ** (−0.15 **)	0.17 ** (−0.04)	0.36 *** (0.26 ***)	0.48 *** (0.49 ***)	0.34 *** (0.29 ***)	1

***Note.*** Outside the brackets are data of left-behind children, and inside the brackets are data of non-left-behind children. Gender was dummy coded such that 0 = boys and 1 = girls. *** *p* < 0.001, ** *p* < 0.01, * *p* < 0.05.

**Table 2:** Results of mediation analyses among left-behind children and non-left-behind children.

Outcome	Predictors	Analysis 1 (Left-Behind Children)					Analysis 2 (Non-Left-Behind Children)				
		*R*	*R* ^2^	*F*	*β*	*t*	*R*	*R* ^2^	*F*	*β*	*t*
Aggressive behavior		0.36	0.13	113.02			0.25	0.06	30.55		
	Neuroticism				0.27	10.63 ***				0.22	5.53 ***
Interpersonal sensitivity		0.56	0.32	222.34			0.51	0.26	160.49		
	Neuroticism				0.57	18.63 ***				0.58	12.57 ***
Bullying victimization		0.32	0.09	39.22			0.20	0.04	9.19		
	Neuroticism				0.13	2.87 ***				0.01	0.13
	Interpersonal sensitivity				0.23	5.32 ***				0.19	3.74 ***
Aggressive behavior		0.53	0.28	96.06			0.53	0.28	57.78		
	Neuroticism				0.08	3.03 ***				0.01	0.04
	Interpersonal sensitivity				0.25	9.08 ***				0.34	9.44 ***
	Bullying victimization				0.15	6.49 ***				0.16	4.93 ***

***Note.*** *** *p* < 0.001.

**Table 3:** The bootstrapping analysis of the mediating effects.

	Analysis1 (Among Left-Behind Children)				Analysis2 (Among Non-Left-Behind Children)			
	Effect	BootSE	95% CI	Relatively Effect	Effect	BootSE	95% CI	Relatively Effect
Total indirect effect	0.18	0.025	[0.13, 0.23]	55.35%	0.21	0.03	[0.16, 0.28]	51.22%
Neuroticism→ Interpersonal sensitivity→ Aggressive behavior	0.12	0.023	[0.10, 0.19]	36.90%	0.17	0.03	[0.14, 0.26]	41.47%
Neuroticism→ Bullying victimization→ Aggressive behavior	0.03	0.008	[0.01, 0.03]	9.23%	0.01	0.01	[−0.02, 0.02]	
Neuroticism→ Interpersonal sensitivity→ Bullying victimization→ Aggressive behavior	0.03	0.007	[0.01, 0.04]	9.23%	0.03	0.01	[0.01, 0.03]	7.32%

## Data Availability

The data presented in this study are available on request from the corresponding author.
